# 3D-printed components for quantum devices

**DOI:** 10.1038/s41598-018-26455-9

**Published:** 2018-05-30

**Authors:** R. Saint, W. Evans, Y. Zhou, T. Barrett, T. M. Fromhold, E. Saleh, I. Maskery, C. Tuck, R. Wildman, F. Oručević, P. Krüger

**Affiliations:** 1 School of Physics and Astronomy, The University of Nottingham, Nottingham, NG7 2RD United Kingdom; 2 Department of Physics and Astronomy, University of Sussex, Brighton, BN1 9QH United Kingdom; 3 Faculty of Engineering, EPSRC Centre for Innovative Manufacturing in Additive Manufacturing, University of Nottingham, Nottingham, United Kingdom

## Abstract

Recent advances in the preparation, control and measurement of atomic gases have led to new insights into the quantum world and unprecedented metrological sensitivities, e.g. in measuring gravitational forces and magnetic fields. The full potential of applying such capabilities to areas as diverse as biomedical imaging, non-invasive underground mapping, and GPS-free navigation can only be realised with the scalable production of efficient, robust and portable devices. We introduce additive manufacturing as a production technique of quantum device components with unrivalled design freedom and rapid prototyping. This provides a step change in efficiency, compactness and facilitates systems integration. As a demonstrator we present an ultrahigh vacuum compatible ultracold atom source dissipating less than ten milliwatts of electrical power during field generation to produce large samples of cold rubidium gases. This disruptive technology opens the door to drastically improved integrated structures, which will further reduce size and assembly complexity in scalable series manufacture of bespoke portable quantum devices.

## Introduction

The current expansion in the field of quantum technologies and in particular quantum sensors has given rise to notable strides forward in device sensitivities with applications ranging from satellite independent navigation to non-invasive biomedical imaging^1,2^. As demand for wider use of these instruments grows, it is imperative to develop robust low-footprint components to provide practical portable systems.

Recent work has focused on the exploitation of quantum phenomena in compact out-of-laboratory devices based on latest advances in micro-manufacturing technology including optical and electron beam lithography^3^, waveguide writing^4^ and reactive ion beam etching^5^. These developments have enabled integrated atom chip based systems^6,7^ and commercial development of complete systems on a vehicle payload scale^8–10^. Additive manufacturing (3D-printing) is a significant emerging technology capable of providing solutions to a range of problems due to the design freedoms^11,12^, the scalable production of individually bespoke components, and weight reduction it facilitates. In this work we demonstrate how this radically different manufacturing approach can be used to reach a step change in performance of practical quantum devices.

Atom-based quantum devices exploiting the properties of thermal vapours^13^, trapped cold atomic clouds^14,15^ and quantum degenerate gases^16^ have been demonstrated experimentally. Many attempts to reduce experimental demands in atom trapping have been considered including single-beam magneto-optical trap (MOT) designs based on conical^17^, pyramidal^18,19^ and tetrahedral^20^ diffraction-based reflectors to reduce optical equipment. Evolving atom chip technology is being pursued for small-scale field production^1,21,22^. Current miniaturised traps used for magnetic trapping within large integrated systems for Bose-Einstein condensate (BEC) production have been conventionally machined^23^ restricting scalability in manufacture.

**Figure 1:**
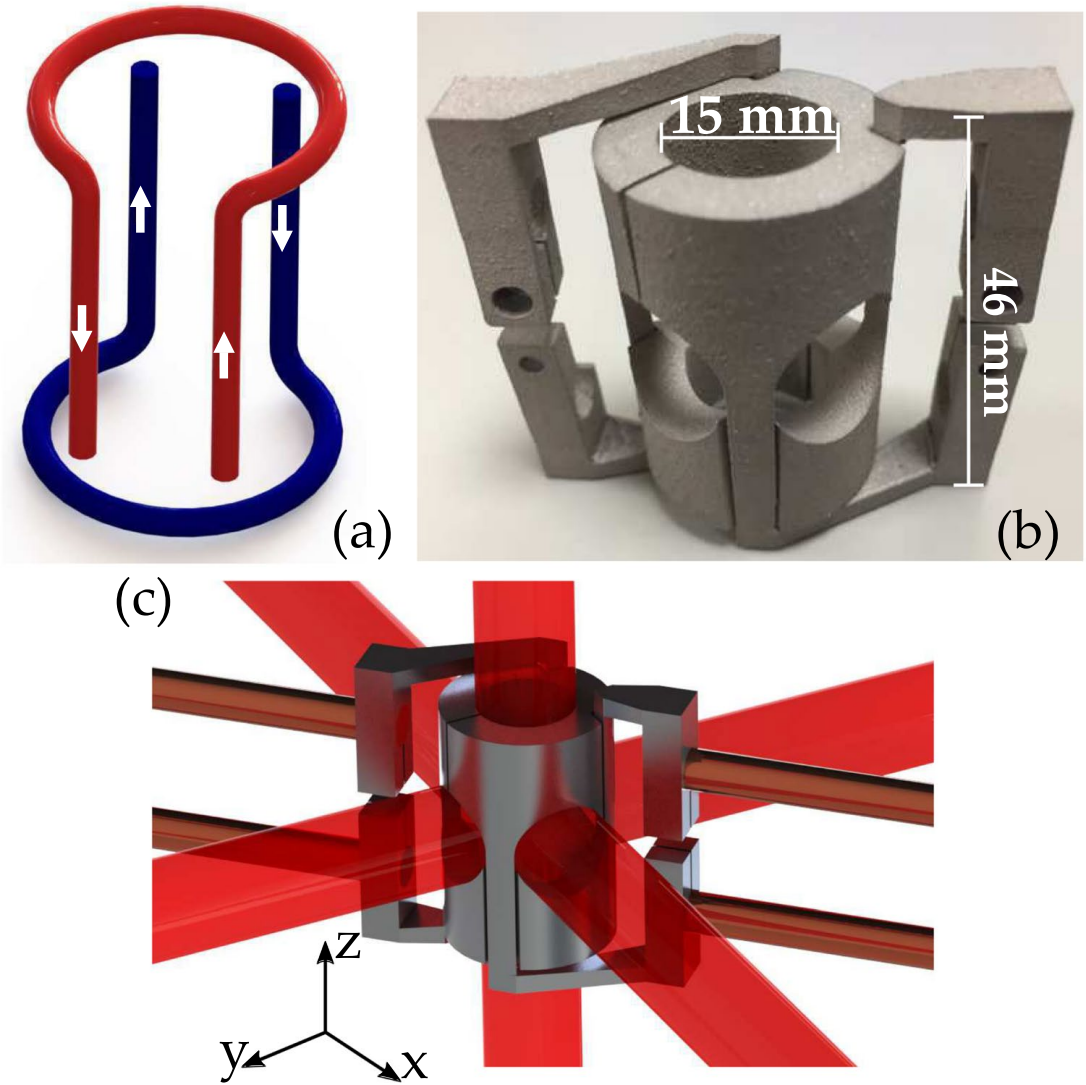
(**a**) Schematic of the cylinder atom-trap current flow. (**b**) 3D-printed atom trap structure. (**c**) Digital render of the cylinder structure shown with vacuum feedthroughs and laser beams.

Here we present (Fig. 1) a 3D-printed centimetre scale demonstrator device providing a quantum resource through production of trapped cold atomic gases with unprecedented efficiency. While power consumption in the high-resistance chip conductors remains large, our approach in contrast allows us to trap tens of millions of atoms with merely 4 mW of electrical power consumption, and with magnetic field switching times under 10 μs. Our ultrahigh vacuum compatible device reliably produces a gas of up to 10^8^ rubidium-87 atoms at a temperature of 20 μK fulfilling the demands of current experimental procedures^19^. We report here the design, manufacture and characterisation of the key performance properties of our prototype.

## Results

### Design Considerations

In the standard experimental implementation of a MOT a pair of coils with parallel orientation and counter-propagating currents (anti-Helmholtz configuration) is used to form linear field gradients with a central field zero as required. While this type of set-up, normally assembled exterior to the vacuum chamber containing the cold atoms, is flexible and robust within a laboratory environment, power consumption remains high and portability is compromised. It is possible to produce a planarised conductor configuration to form a quadrupole field with an out-of-plane zero^24^. This allows for standard chip implementation. Such two-dimensional (2D) designs, however, are at a clear disadvantage in their power-efficiency when compared to three-dimensional (3D) implementations.

Details of a simple comparative model showing a substantial increase in power consumption for 2D and 3D configurations are shown in the supplementary material. The argument can be generalised to any use of magnetic fields for trapping and manipulation of cold atoms, where field minima need to be created away from field-producing structures. Typically a design goal is to reduce power, especially for portable device implementations. Miniaturisation of components generally has the advantage of reducing power consumption, where the ohmic power dissipation overall scales as *P* = *ZI*^2^ ~ *R*^3^, i.e. with the *volume* of the device. It should be noted that for heat management, sometimes *power density* is primarily relevant instead, which is size independent. At a given device size optimal use of the available volume for conductors reduces both power and power density.

MOT devices must give sufficient space for optical pathways and so rely on either larger-scale 3D structures without sufficient design freedom for optimisation^25^ or on miniaturised complex 2D chip-geometries^1^. The considerations discussed show that an ideal MOT device would be based on a miniaturised 3D structure with optimal use of the available volume, which is typically defined by other constraints, such as access to laser beams for use in atom cooling, detection and sensing. Here additive manufacturing allows for these design constraints to be simultaneously met.

### Prototype Manufacture

Additive manufacturing paired with computer aided design (CAD) allows for robust and almost arbitrarily shaped designs. The model case of our compact demonstrator illustrates the possibility to fully and simultaneously implement the aforementioned design rules. A schematic of our chosen electric current path which creates a quadrupole magentic field is shown in Fig. 1(a). The four central parallel conductors carrying currents with alternating flow directions form the two-dimensional quadrupole field. The translational symmetry is broken by counter-propagating current loops in two parallel planes orthogonal to the four straight conductors. The parameters of the geometry, in particular the ratio of the length of the straight conductors to the radius of the current loops, determines the gradient ratio of the emergent magnetic field^26^. Complex geometries, such as the cylinder presented here are challenging to manufacture with traditional methods. Consequently, they are often assembled from smaller and simpler parts, which can introduce contact resistances and parasitic capacitances that harm field generation and decay times. In contrast, the design freedom and flexibility provided by additive manufacturing allows for such structures to be produced as monolithic components.

CAD is used to create an experimentally viable design with a sizeable trapping region, optical access, structural supporting arms and electrically conductive contacts. Fitting these constraints we produce a centimetre scale (35 cm^3^) cylindrical device [Fig. 1(b)] forming a trapping region of 1.8 cm^3^. The overall volume of the device is chosen to be small for reasons of power consumption while still being large enough to permit laser beams with sufficient diameter to enter the central trapping region. We include electric feeds that simultaneously serve as supporting arms with clamping connections to commercially standard CF40 electrical feedthroughs. Insulation between the two opposing currents is implemented by manufacture of two separate monolithic components. The design volume is maintained where possible to maximise current carrying material and mitigate resistive heating. Design prototypes can be easily passed onto finite element packages for calculation of the resultant magnetic field, creating a responsive feedback loop between design and field simulation. Our final printed structure is displayed in Fig. 1(b), henceforth referred to as the *cylinder trap*, and a digital render of the experimental system is shown in Fig. 1(c).

**Figure 2:**
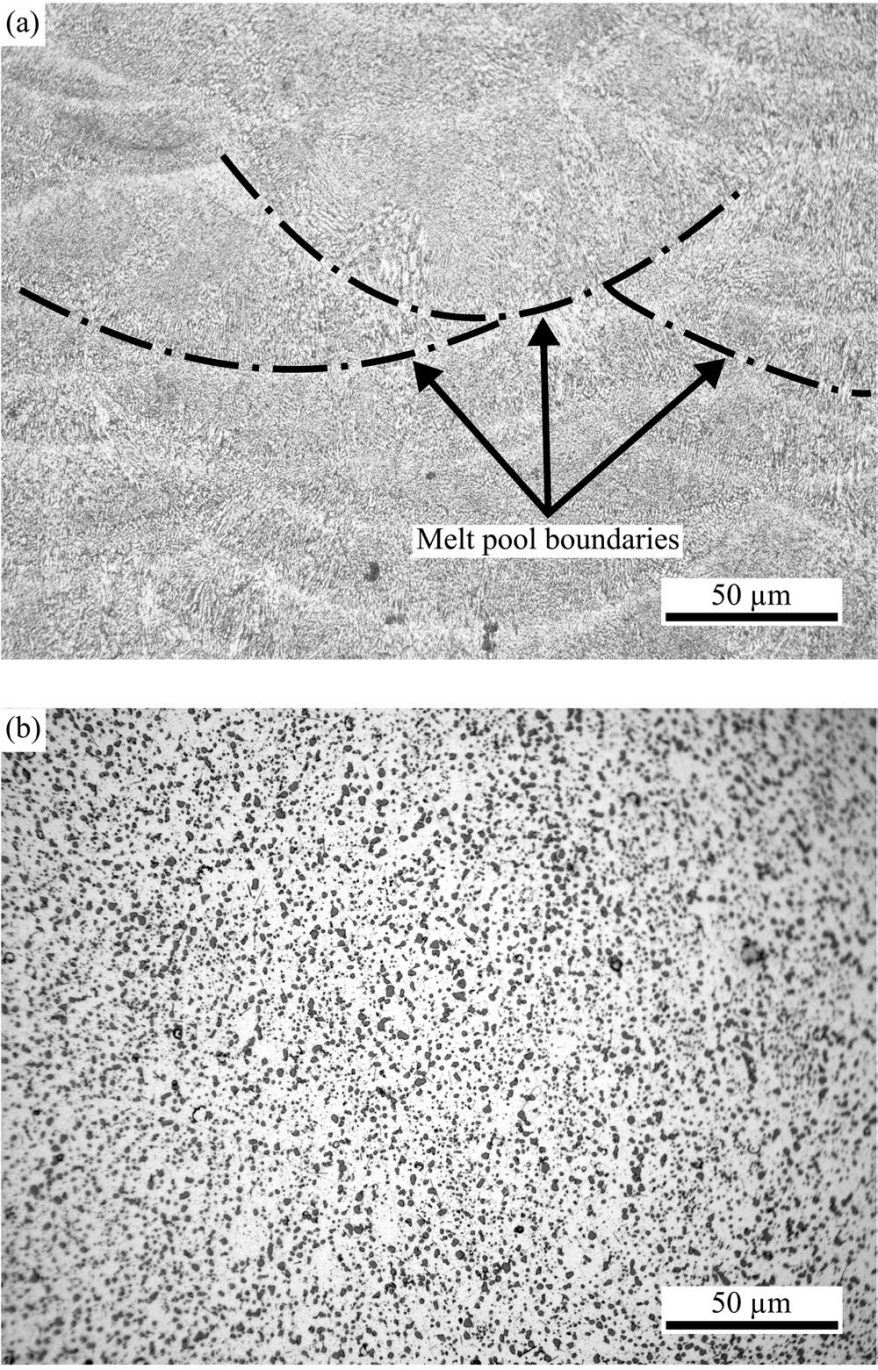
Typical surface pattern images from an optical microscope. (**a**) Untreated structure showing the material immediately after manufacture; the darker grey regions separated by lighter channels are melt pools formed during selective laser melting (SLM). (**b**) Images following solution heat treatment showing the more uniform distribution of silicon pools in dark grey. The scale bar depicts 50 μm.

The additive manufacturing technique we employ for 3D-printing this device is selective laser melting (SLM) of an Al-Si10-Mg alloy^27^. This method is based on scanning a high-power laser beam over a bed of powdered metal. In addition to the standard technique, we apply solution heat treatment (SHT), which is usually carried out to improve mechanical properties. Here it enhances the electrical conductivity through significant changes in the microstructure, as shown in Fig. 2. The heterogeneous layers produced during the SLM process are reorganised and form a homogeneous distribution of silicon particulates within an aluminium matrix. The resulting conductivity reaches 70% of the bulk value, making Joule heating effects in the material negligible in all our experiments. The microscopic origin of this improvement is currently under investigation. In contrast to improved conductivity through densification^28^, the SHT method implemented here has no effect on the porosity of the material^29^. Further details are found in the methods section. The device manufactured in this way is compatible with ultra-high vacuum conditions.

An off-the-shelf compact vacuum system houses the cylinder trap and is pumped down to ~10^−7^ mbar with a turbomolecular pump. Ultra-high vacuum (<10^−10^ mbar) is then achieved after baking the chamber at 200 °C and pumping via a passive non-evaporable getter pump and a compact 6 l/s ion pump. UHV conditions were maintained for the entire nine month duration of the experiment with no significant outgassing detected, as confirmed by the pressure continuously remaining below our measurement threshold of 10^−10^ mbar. Although similar results have been acheived with titanium and silver samples^30,31^ this is an untested characteristic of 3D-printed Al-Si10-Mg.

### Performance Data

**Figure 3:**
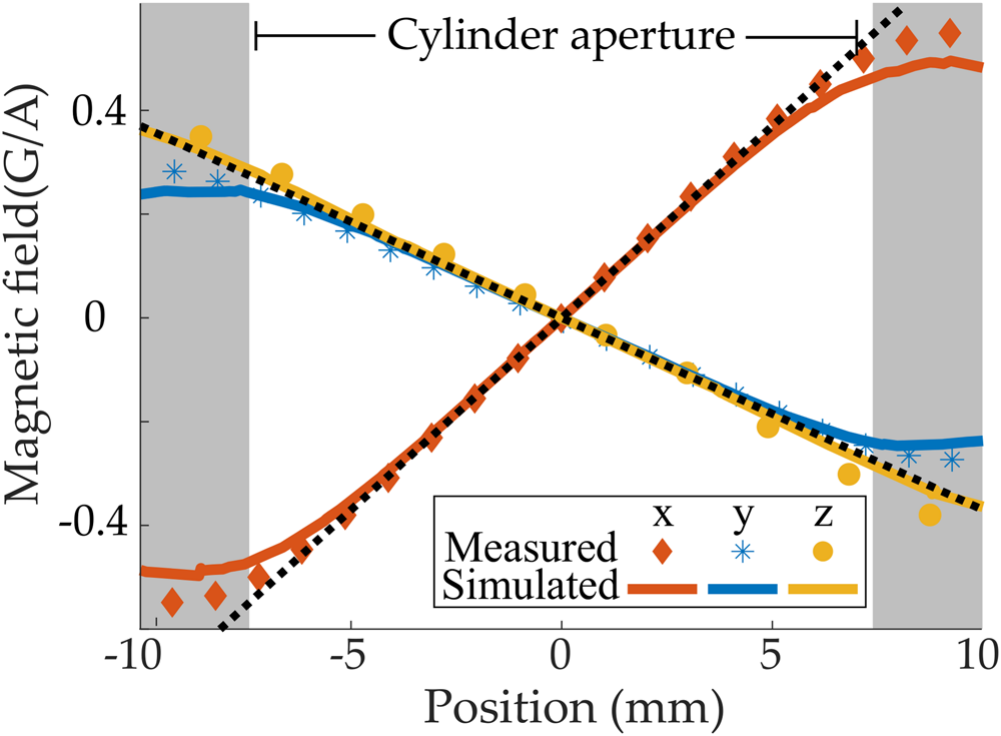
Plot of the calculated (finite-element) versus measured magnetic field magnitude along each axial direction. Dotted lines here show ideal 2:−1:−1 ratio between strong and weak axes. Along all three optical axes, the linear field region extends over the full cylinder aperture, ensuring optimal laser cooling.

A trapping field gradient of ~10 G/cm along the strong axis is typical of a MOT using rubidium-87. Field gradients across the trapping region are linear in the ideal case. The finite element calculated magnetic field profiles are shown in Fig. 3 alongside measured values of the field strength. The fields produced by our prototype closely fit both criteria and the field geometry produced is that expected of the anti-Helmholtz configuration with a −2:1:1 ratio between strong and weak axes. A gradient of 10 G/cm is produced at a manageable current of 14 A. Measurements at currents up to 50 A with corresponding gradients of up to 37 G/cm have been performed, confirming the expected linear relation between gradient and current and the wide range of control of the device within the regime of negligible heating. The switching time of quantum devices is important for their performance. In traps using magnetic fields the switching time is normally limited by eddy currents that persist in neighbouring metallic material, increasing the field decay times to typically several milliseconds. Here, there are no structures within the device to host eddy currents and the intrinsically low inductance of the device ((0.49 ± 0.05) μH) allows the magnetic fields to be removed within (13.0 ± 2.3) μs as measured directly with a Hall probe measurement (see supplementary material for more details).

**Figure 4:**
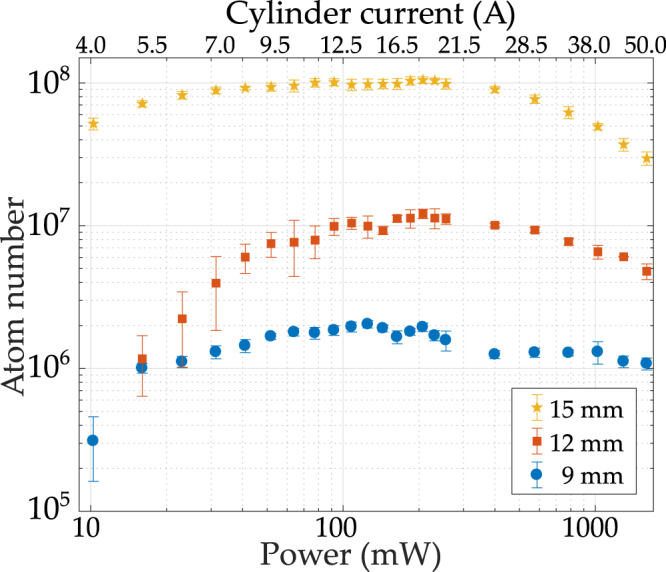
Maximum atom number as a function of cylinder electrical power dissipation (current) for three beam diameters. The current range 4 A to 50 A corresponds to the magnetic field gradient range 3.2 G/cm to 40 G/cm.

We can quantify the performance of our cold-atom source by correlating the power consumption of the device to the achieved atom number. The required cloud size is dependent on system use. In our demonstration experiments we aimed to produce 10^7^ atoms corresponding to the standard set in existing thermal atom quantum devices^8^. We find that our device is even capable of producing a cloud of more than 10^8^ atoms as necessary for Bose-Einstein condenstate production^22^. Measurements are taken of the atom number, via absorption imaging, for varying cylinder currents *I* and corresponding power consumptions *P* = *ZI*^2^ with the Ohmic device resistance *Z*, and are shown in Fig. 4.

First we consider the capabilities of the device by measurement of the electrical power across the cylinder itself with a maximal beam diameter of 15 mm. Atom numbers exceeding 10^8^ are reached for power levels of 30 mW and above (the typical gradient of 10 G/cm corresponds to ~100 mW), with a broad plateau and atom numbers dropping by a factor of 2 at 10 mW power consumption and below. Even down to the lowest measured power of 4 mW the atom number stayed well above 10^7^. Quoted power consumptions are based on the cylinder resistance, but even when considering all connecting leads the power increased by less than 50%. With regards to standard atom traps, such as coil systems mounted externally to the chamber or macroscopic intra-vacuum conductor assemblies in BEC experiments, which can require up to 10 W of power, a power reduction of orders of magnitude is found for our prototype. This confirms the scaling of power with device volume discussed above, i.e. the cubic scaling of power with a characteristic length scale of the device.

The above conceptual considerations supported by the experimental findings obtained with a small volume device suggest that further miniaturisation could lead to ever decreasing power consumption. It has, however, been found that laser cooling with small beam diameters becomes inefficient. The exact scaling of the atom number N with beam diameter is largly dependent on the experimental system, but strong power laws have been verified, e.g. *N* ~ *D*^5.82 ± 0.05^ ^32^. Leaving clear apertures for sufficiently large laser beams is therefore a constraint that limits the drive towards smaller volume trapping assemblies. The size of the optimal apertures depends on the target atom number used in e.g. the employed quantum sensing scheme. For our prototype we chose the 15 mm diameter aperture to guarantee atom numbers in excess of 10^7^ − 10^8^ while still keeping power levels in the lower mW range.

In order to empirically explore whether even lower apertures are viable - and consequently whether a significant reduction in electrical power consumption (scaling with the cube of the aperture) can be achieved - we performed measurements with reduced cooling laser beam diameters (Fig. 4). We begin with Gaussian beams with 1/e^2^ diameters of 2 inches (50.8 mm) then truncate these with iris diaphragms to a given diameter. Following a 20% reduction of the beam diameter from 15 mm to 12 mm the device still yields more than 10^7^ cold atoms. In an aperture size-adjusted prototype this would now be achieved at 50% of the original power level (in our simulated case we use ~200 mW). A further beam diameter reduction to 9 mm (60% of the original size) allows for atom numbers of (2.0 ± 0.1) × 10^6^. A size-adjusted device would produce the same gradients as our manufactured prototype at merely 20% of the power, in this case. Measurements for a range of current and power levels in our system (ranging between 10 mW and 1.6 W) are shown in Fig. 4. For each cylinder current the cooling light is detuned to maximise atom number in accordance with the dependence of the applied current to the field gradient^33^. The peak red-detuning varies from 16 → 25 MHz for currents 4 → 25 A. In all cases the atom number reaches a plateau at currents of ~10 A, corresponding to field gradients along the strong quadrupole axis of ~7 G/cm. Operation at high atom numbers is possible over a wider range of gradients (~7–20 G/cm) with only gradual reduction for smaller (and larger) gradients.

Finally, a key property of a useful quantum resource is the acheived temperature of the cloud, as colder atoms can enhance the performance of sensors depending on the protocols being used^34^. In our experiment, temperatures are determined using the standard time-of-flight (TOF) imaging technique^35^, whereby the cloud is expanded ballistically for various times after switching off the trapping potential. A weak resonant imaging beam is directed along the *z*-axis, as shown in Fig. 1(a), and the density distribution is recorded using a charged-coupled device (CCD) camera, allowing measurement of the momentum distribution from which the temperature is inferred^36^.

**Figure 5:**
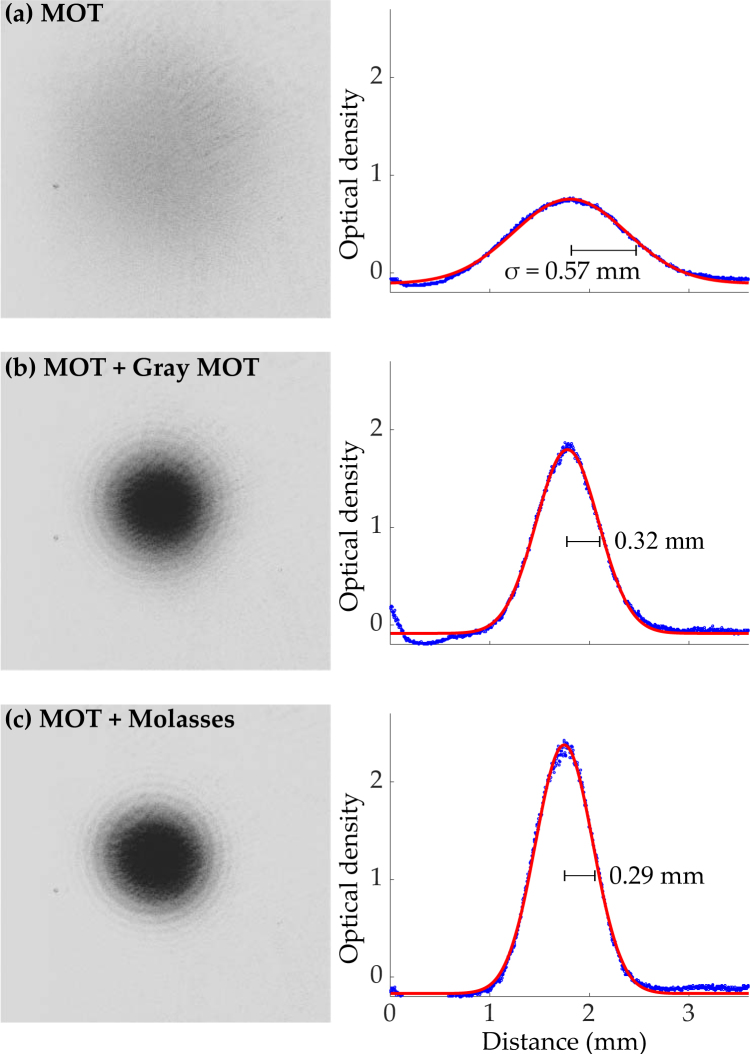
Left: Optical density images of a cloud of atoms in three cooling regimes. Each single shot measurement is taken after 12 ms time of flight. Right: Vertically integrated optical densities (blue) with superimposed Gaussian fits (red). (**a**) A typical MOT cloud, (**b**) a MOT cloud after Gray MOT cooling (see text), (**c**) a MOT cloud after molasses cooling. The Gaussian width *σ* is indicative of the cloud temperature.

Without any additional cooling stages we produce a MOT with a temperature of (170 ± 4) μK, an example optical density image of which is shown in Fig. 5(a). This temperature is close to the Doppler cooling limit for rubidium, 143 μK. Reducing the temperature further, Fig. 5(b) shows an example optical density image taken following a simple ‘Gray MOT’ sub-Doppler cooling sequence^37^ carried out at the end of MOT loading. Here light is detuned while the magnetic fields are kept active. A ramp of 6 μs to a maximal red-detuning of 10Γ (Γ being the natural line width of the transition Γ = 6.065 MHz) is used here to yield a cloud of (3.7 ± 0.2) × 10^7^ atoms at a temperature of (29.1 ± 1.2) μK, where the error corresponds to the standard deviation from statistics over many experimental runs. Even lower temperatures can be achieved by implementing a molasses cooling scheme involving a 3.2 μs ramp to a maximal red-detuning of 10Γ after MOT loading, with no magnetic fields. Figure 5(c) shows a cloud of (4.0 ± 0.2) × 10^7^ atoms at a temperature of (20.1 ± 0.2) μK as a result of applying this scheme. This scheme is less robust than a Gray MOT cooling scheme with respect to optical alignment and ambient magnetic field control. In practice the modest improvement in cooling for the molasses over the Gray MOT cooling does not justify the loss of robustness of the scheme.

## Discussion

We have introduced additive manufacturing as a key technology for a new generation of quantum devices and device components. To illustrate some of the central capabilities of this new approach we designed, built and tested a compact cold atom source. In an efficient prototyping cycle, we are able to convert schematic field-generating structures under consideration of design rules and constraints into full 3D CAD files. These files directly interface with standard finite-element software for further design iteration and optimisation and then serve as direct input for prototype manufacture on a 3D-printer. A simple postprocessing procedure allows us to overcome common challenges associated with 3D-printed conductor structures in terms of vacuum compatibility and low conductivity. This opens the path towards exploiting the full potential of additive manufacturing for quantum devices over a wide range of applications.

Using the freedom of design and flexibility of geometry possible with additive manufacturing, we demonstrate that our prototype functions as a ready-to-use cold atom source module for quantum sensors. The ability to produce >10^7^ cold atoms makes this technique a prime candidate to replace the standard quantum resource in thermal atom systems. The further capability of providing large samples of >10^8^ cold atoms paves the path towards a simple quantum gas source in devices using Bose-Einstein condensates (BECs) as resource. In addition to the robustness, ease of manufacture and implementation of any design variations in the prototyping, a particular appeal of this device is its efficiency, with power consumption reaching levels below 10 mW. This extreme reduction in power consumption of the trapping structure minimises the required resources for the system as a whole and with the rapidly progressing technological developments of associated experimental systems and evolving 3D-printing techniques significant further system integration is expected. Future designs could incorporate mounting structures for optical components following trends in minimised optics systems or allow for integration with 2D-MOT systems or atom chip technology to allow for alignment with BEC experimental procedures. Emerging capabilities of additively manufacturing multi-material components will provide crucially important means of full compact systems integration.

## Methods

### Production Details

A Renishaw AM250 selective laser melting (SLM) machine is used to produce Al-Si10-Mg samples from a powder-alloy of chemical composition Al 88.9 wt%, Si 10.7 wt%, Mg 0.5 wt% (particulate sizes 15 μm to 100 μm)^27^. Structures are created by melting the successive layers of powder with a 200 W Yb-Fiber (*λ* = 1064 nm) laser. Current 3D-printing methods are capable of manufacture with various alloys of titanium, steel, stainless steel and silver. Here Al-Si10-Mg is used for the convenient electrical properties and low cost.

Once printed, the structure is heated at 520 °C for 1 hour, followed by water quenching and artificial aging at 160 °C for 6 hours with both steps carried out in a pre-heated furnance with an air atmosphere^38^. This process yields a cold resistivity of 5 × 10^−8^ Ωm or a resistance of (640 ± 4) μΩ for our geometry which is a 20% reduction from the as-printed structure resistance of (800 ± 20) μΩ. A direct comparison of pure aluminium^39^ puts the conductivity of this Al-Si10-Mg alloy at 70% of the value for bulk material at room temperature. The dominant cooling mechanism of the mounted device is thermal conduction through the electric feedthrough simultaneously serving as mechanical mount. The body of the vacuum chamber serves as heat sink. As convection (air cooling) is irrelevant in this set-up, it is sufficient to measure heating effects under atmospheric conditions. We performed measurements for currents of up to 50A, with the hottest-point temperature (located at the corner of the cylinder arm) never exceeding 36 °C.

### Light Frequency Manipulation

Our optical cooling field is generated by six independent beams created by a diode laser locked to the ^87^Rb D2 line, 5^2^*S*_1/2_ → 5^2^*P*_3/2_ hyperfine *cooling transition* (F = 2 → F′ = 3) providing a per-beam power of 40 mW distributed across Gaussian beam with 1/e^2^ diameters of 2 inches (50.8 mm), which are truncated with iris diaphragms to the diameter of the optical access ports of the atom trap. This results in an actual per-beam power of 8 mW with a peak intensity of 4 mW cm^−2^ (corresponding to over saturating the transition by a factor of 2.5). The light frequency is red-detuned by 20 MHz (~three times the natural line width) from the cooling transition.

To close the transition, a small amount of additional light at the *repumping transition* F = 1 → F′ = 1 is required. We derive this light from a separate diode laser, whose light is coupled into one of the three fibre pairs feeding the light to the trapping region. A resonant imaging beam (F = 2 → F′ = 3) is then coupled into one of the vertical beams (*z*-axis) to generate absorption images^36^, [3.2] of the various atom clouds. Our fully fiberised set-up is immediately ready for a replacement of our commercial laser sources by integrated miniature systems as are becoming readily available at the moment^40^.

### LIAD Rubidum Desorption

A ^87^Rb background pressure is generated via current flow through evaporative dispensers. Aditionally, a light induced atomic desorption (LIAD)^41^ ultra-violet LED system is triggered in the experimental sequence. By using LIAD only during the loading phase of our MOT we are able to achieve a low average background pressure of rubidium in the chamber, as well as reducing the required dispenser current. This process can also improve the lifetime of any magnetic traps added to later designs.

### Experimental Control System

The control systems of cold atom experiments have traditionally relied on expensive hardware running bespoke user created software or scripts developed in environments such as C++ and LabVIEW which require significant expertise in software development. To provide a low-power, small-volume (1.2 l) and low-cost portable solution, our experimental control is implemented in a master-slave configuration comprising of three micro-controllers. One *master* micro-controller provides 12 fast digital transistor-transistor logic (TTL) signals, two of which are used to communicate to two *slave* devices, each providing two analogue channels. We reduce the minimum on-off pulse width from 5 μs to 100 ns using low-level code changing specific bits on the microprocessor rather than using slower high-level if-statement-based development environment functions. A compact battery array can service the three 5 V micro-controllers and attached simple 12 V step-down and amplification circuits. The low-cost hardware and accessible high-level integrated development environment (IDE) allows for fast and flexible design and prototyping of experimental sequences for the trapping and cooling of atoms.

### Data Availability

The datasets generated during and/or analysed during the current study are available from the corresponding author on reasonable request.

## Electronic supplementary material

Supplementary Material
