# Supplementary material for: 3D-printed components for quantum devices
Source: Sci Rep. 2018 May 30;8:8368. doi: 10.1038/s41598-018-26455-9 (PMC5976634; doi:10.1038/s41598-018-26455-9)
Supplement: Supplementary file 1 — Supplementary Material [file 41598_2018_26455_MOESM1_ESM.pdf]

Supplementary Material - 3D-printed components for quantum devices

R. Saint,[1,2] W. Evans,[1,2] Y. Zhou,[1] T. Barrett,[1,2] T. M. Fromhold,[1] E. Saleh,[3]
I. Maskery,[3] C. Tuck,[3] R. Wildman,[3] F. Oručević,[1,2] and P. Krüger[1,2]

[1]*School of Physics and Astronomy, The University of Nottingham, Nottingham, NG7 2RD, United Kingdom*
[2]*Department of Physics and Astronomy, University of Sussex, Brighton, BN1 9QH, United Kingdom*
[3]*Faculty of Engineering, EPSRC Centre for Innovative Manufacturing in Additive Manufacturing, University of Nottingham, Nottingham, United Kingdom*

(Dated: 26th April 2018)

Supplementary Note 1 - Device model

A simple idealized comparison of a three-dimensional (3D) versus two-dimensional (2D) scenario of generating a quadrupole field demonstrates the key principles behind beneficial power consumption. We consider a planarised conductor configuration forming a quadrupole field with an out-of-plane zero formed by multiple in-plane currents, whose fields compensate each other at the zero position. As all these fields necessarily drop monotonically in magnitude with distance from the plane, the field gradients will also at least partially compensate each other at the field zero. This gradient compensation needs to be minimised in order to obtain a power-efficient planar solution. In contrast in 3D the currents generating the fields that cancel at the trap center position of a device can be formed in such a way that the gradients produced by them add, rather than subtract as in planar implementation.

As a simple model illustrating the 2D vs 3D difference, we choose two infinitesimally thin current loops in the anti-Helmholtz configuration (two parallel loops with equal radius R carrying equal currents I are placed at a distance $d = R$ from each other) and compare them to two in-plane concentric current loops (Supplementary Figure 1). The in-plane loops have radii R_1 and R_2 and carry currents I_1 and I_2, respectively. The 3D configuration field zero occurs at a distance $R/2$ from either loop. Consequently, the planar configuration parameters are chosen such that a field zero forms at $R/2$ from the current plane in that case. We further impose equal power consumption in both configurations which is obtained when $R_1 {I_1}^2 + R_2 {I_2}^2 = 2RI^2$. Finally, we require the field curvature to vanish at the field zero, so that in approximation of the ideal quadrupole field the field varies only linearly at this center position. Note that without loss of generality it is sufficient to only consider the field along the loops' symmetry axis z, where the field is always oriented along z. We find that under the above constraints the maximal gradient is achieved in the planar configuration when $R_1 = 1.14\,R$ and $R_2 = 2.51\,R$ with the currents $I_1 = 0.46\,I$ and $I_2 = -0.84\,I$. Even in this optimal configuration, the gradient is reduced by a factor larger than 7 with respect to the 3D anti-Helmholtz configuration. Calculated field configurations are shown in Supplementary Figure 1(a) for a 3D structure and (b) for a planar structure. Supplementary Figure 1(c) displays the corresponding fields along the symmetry axis of the loops. To match the gradient obtained in the 3D configuration with the 2D configuration requires a more than 50-fold increase of power consumption.

Now let us examine the relationship between device size and power consumption. Again, for the 3D case, the idealised anti-Helmholtz configuration may serve as an illustration of a scaling law that is extendible to more general magnetic field generating structures. In the anti-Helmholtz configuration, as in other configurations e.g. for magnetic traps of various shapes, the key parameter is the generated field gradient at the field zero (field minimum). As the field of a single current loop at the position of the quadrupole field zero ($z = R/2$) scales as $\sim 1/R$, the gradient at that position scales as $\sim 1/R^2$. Conversely, in order to maintain a constant gradient, the required current scales as $\sim R^2$. If the conductor cross section is assumed to scale with size of the device, the resistance Z of the structure increases with the length of the current loop ($\sim R$) and drops as $\sim 1/R^2$ with the cross section, such that the Ohmic power dissipation scales as $P = ZI^2 \sim R^3$. This signifies that power consumption will drop cubically as we scale down the device with a characteristic radius.

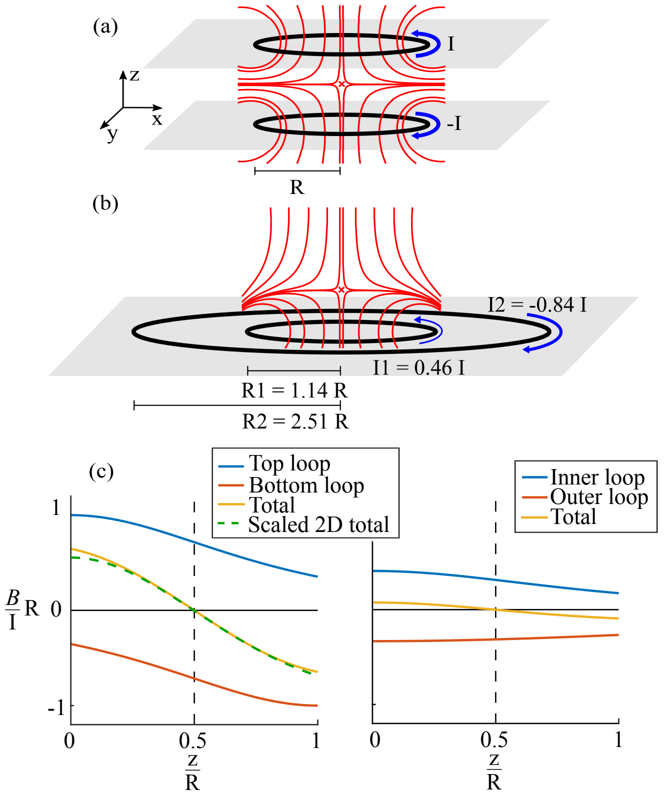

FIG. 1. Comparison of two quadrupole field generating structures whereby two current loops are placed in two parallel planes (3D) or a single plane (2D). Streamline plots show the field produced from the 3D system (a) and 2D system (b). (c) Magnetic field strength along the symmetry axis z of the current loops (due to symmetry radial fields vanish). The field of one loop (blue) is compensated by the other (red) in both configurations at the same zero-field position (red cross). At equal power consumption, the total field gradient (yellow) for the 3D system is stronger than that in the 2D case by a factor of 7.37. To reach the same gradient with the planar 2D assembly, the current needs to be scaled up by the same factor (dashed green line). This corresponds to an increased power dissipation by a factor of 54.3.

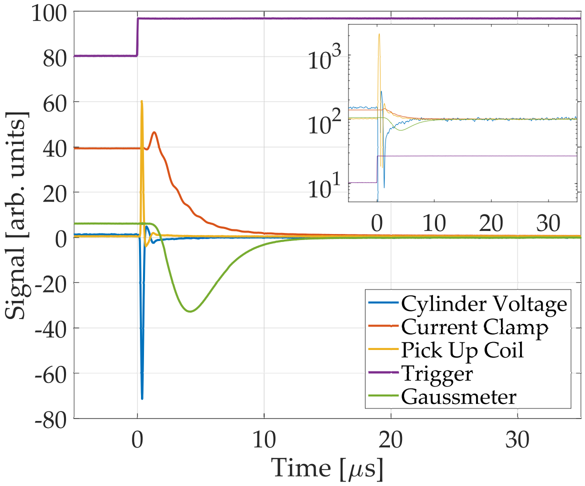

FIG. 2. Magnetic field decay measurement. The plot shows the characteristics of the switch-off process for the cylinder, using a current of 15 A. The voltage across the device itself (blue trace) is displayed, along with the current flowing via a current clamp (red), after sending a trigger (purple) to open an IGBT. Also shown is the voltage induced in a pick-up coil due to changes in the magnetic field (yellow), along with the signal from a Hall effect Gaussmeter (green). After an initial transient period, the Hall probe signal decays to below 10 % of its initial value within (13.0 ± 2.3) µs, after which time all signals settle to their steady state background readings. The inset depicts the same data set on a semi-log plot to emphasise the similar decay times of all measured signals.

Supplementary Note 2 - Field Decay Measurement

It is important to investigate the decay of the magnetic field after switching off the trap as this can limit the experimental cycle length in typical cold atom devices.

To characterise this switching process, the voltage across the cylinder was measured along with the current flowing through it using a current clamp (Chauvin Arnoux P01120043A), as a function of time after opening an insulated gate bipolar transistor (IGBT). In addition, the magnetic field inside the cylinder during the switching was measured using a Hall effect Gaussmeter (Hirst GM08) and its derivative with a small single-turn pick-up coil. Both the Hall probe and the pick-coil were oriented along the strong eigenaxis of the quadrupole field, at the position of largest field. The results are shown by the signal traces in Supplementary Figure 2, obtained for a current of 15 A, corresponding to a magnetic field of (6.2 ± 0.6) G at the position of the Hall probe.

Shortly after opening the IGBT, a large flyback voltage develops across the cylinder, as expected when switching a current through an inductive load. Some oscillatory behaviour can also be seen, arising due to contact resistances and small parasitic capacitance and inductance within the circuit, which are non-negligible in comparison to the impedance characteristics of the cylinder itself. Following the initial transient period of the switching process, the magnetic field measured with the Hall probe is seen to decay below 10 % of its initial value within (13.0 ± 2.3) µs.

Finally, in order to determine the inductance, L, of the cylinder the resonant frequencies, $f_{\mathrm{res}}(C)$, of a parallel LC circuit for various known capacitances, C, were measured with a network analyser (Mini Radio Solutions miniVNA). Supplementary Figure 3 shows a plot of ${f_{\mathrm{res}}}^2$ against $1/C$, and using the relation ${f_{\mathrm{res}}}^2 = 1/(4\pi^2 LC)$, a value for the inductance of (0.49 ± 0.05) µH is extracted from the gradient of the linear fit.

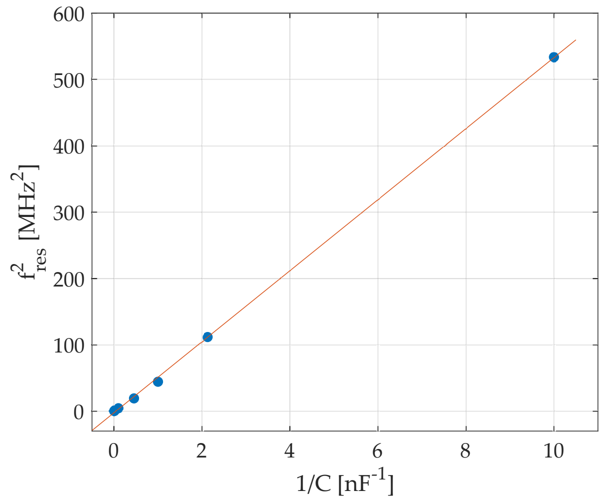

FIG. 3. Cylinder inductance measurement. A plot showing the resonant frequencies, $f_{\mathrm{res}}(C)$ of a parallel LC circuit for various known capacitances, from which an inductance of (0.49 ± 0.05)µH is extracted from the linear fit, as described in the text.
